# Sunlight Control of Interfacial Magnetism for Solar Driven Spintronic Applications

**DOI:** 10.1002/advs.201901994

**Published:** 2019-10-26

**Authors:** Yifan Zhao, Shishun Zhao, Lei Wang, Ziyao Zhou, Junxue Liu, Tai Min, Bin Peng, Zhongqiang Hu, Shengye Jin, Ming Liu

**Affiliations:** ^1^ Electronic Materials Research Laboratory Key Laboratory of the Ministry of Education and International Center for Dielectric Research School of Electronic and Information Engineering and State Key Laboratory for Mechanical Behavior of Materials Xi'an Jiaotong University Xi'an 710049 China; ^2^ International Joint Laboratory for Micro/Nano Manufacture and Measurement Technology Xi'an Jiaotong University Xi'an 710049 China; ^3^ Center for Spintronics and Quantum System State Key Laboratory for Mechanical Behavior of Materials School of Materials Science and Engineering Xi'an Jiaotong University Xi'an Shaanxi 710049 China; ^4^ State Key Laboratory of Molecular Reaction Dynamics and Collaborative Innovation Center of Chemistry for Energy Materials (iChEM) Dalian Institute of Chemical Physics Chinese Academy of Sciences 457 Zhongshan Rd. Dalian 116023 China

**Keywords:** ferromagnetic resonance, low power, magnetoelectric coupling, organics, solar cells

## Abstract

The inexorable trend of next generation spintronics is to develop smaller, lighter, faster, and more energy efficient devices. Ultimately, spintronics driven by free energy, for example, solar power, is imperative. Here, a prototype photovoltaic spintronic device with an optical‐magneto‐electric tricoupled photovoltaic/magnetic thin film heterojunction, where magnetism can be manipulated directly by sunlight via interfacial effect, is proposed. The magnetic anisotropy is reduced evidenced by the out‐of‐plane ferromagnetic resonance (FMR) field change of 640.26 Oe under 150 mW cm^−2^ illumination via in situ electron spin resonance (ESR) method. The transient absorption analysis and the first‐principles calculation reveal that the photovoltaic electrons doping in the cobalt film alter the band filling of this ferromagnetic film. The findings provide a new path of electron doping control magnetism and demonstrate an optical‐magnetic dual controllable logical switch with limited energy supply, which may further transform the landscape of spintronics research.

## Graphical abstract

Visible light control of magnetism is a brand‐new area, and can offer an alternative, straightforward, and efficient way of optical control of spin dynamics, and opens an avenue for applications in low‐power, visible light controlling magnetic devices. As an outlook, the photoinduced magnetic anisotropy change can lead to visible light controllable spintronic devices without high‐power consumption or femtosecond laser instrumentation.

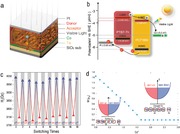

## 1 Introduction

For centuries, researchers have been devoting their lifetime efforts to making electronics cheaper, more compact, and more energy‐efficient.^1^, ^2^, ^3^, ^4^, ^5^, ^6^, ^7^, ^8^, ^9^ A good example is that spintronics applications are the minimization of electronic devices.^1^, ^2^, ^3^, ^10^ Here, spins, instead of electrons, serve as information storage media, as well as logical and information processing devices. Magnetic‐field (H‐field) has been widely used to regulate spins, however, magnetic field is gradually replaced by current or electric field (E‐field) due to its high energy consumption. For example, large magnetoelectric tunability has been achieved in E‐field control of magnetism.^6^, ^11^, ^12^, ^13^, ^14^, ^15^, ^16^, ^17^ Nevertheless, the potential of the present current‐ or voltage‐driven spintronics has still been constrained by the electric power supply network. In other words, novel energy‐independent spintronics/electronics are in great demand.

Considering the universality of the solar power, we then propose a photovoltaic spintronics demo, combining the cutting‐edge technologies from both spintronics and photovoltaic communities, and allowing sunlight manipulation of spin behavior, for the ultimate solution‐solar‐driven spintronics. One of the critical challenges of realizing solar‐driven photovoltaic spintronics is to control ferromagnetism directly with visible light at room temperature (RT).

Nowadays, all‐optical control of ferromagnetism relies on intercoupling photons and spins.^18^, ^19^, ^20^, ^21^, ^22^, ^23^ In ferromagnetic (FM) films, researchers have switched magnetization between two states by femtosecond circularly polarized laser pulses due to the magneto‐optical Faraday effect or optically induced electron and thermal excitations.^18^, ^19^, ^20^ Nevertheless, regarding its applications, the laser‐induced heating close to the Curie temperature could result in unstable optical‐magnetic modulation and high energy consumption, and could also influence the neighbor magnetic domain of optical‐driven memories, which will then limit the storage density and stability. Li et al. realized a 0.24% magnetoresistance change in perovskite La_1/2_Sr_1/2_MnO_3_ at 240 K via light illumination.^21^ Náfrádi et al. manipulated ferromagnetic order in perovskite CH_3_NH_3_(Mn:Pb)I_3_ at ≈5 K with a light induced Ruderman–Kittel–Kasuya–Yosida interactions.^22^ Sun et al. reported a spin current tunable NiFe/C_60_/AlO*_x_*/Co spin vale junction device, in which C_60_ served as a photovoltaic layer.^23^ Nevertheless, these methods may suffer from defects like energy‐extensive consumption, low temperature, structure dependence, or limited optical‐magnetism coupling.

Enlightened by the recent progress of organic semiconductor solar cells (OSSCs) and ionic liquid (IL) charge doping modulation of interfacial ferromagnetism, we have designed a photovoltaic spintronics demo with optical‐magnetic‐electro (OME) tricoupled OSSC/magnetic heterojunction to overcome the current challenges of optical control of magnetism.^11^, ^24^, ^25^, ^26^, ^27^, ^28^, ^29^, ^30^, ^31^, ^32^, ^33^ Here, the OSSC films under visible light can provide a large number of photo‐induced electron accumulation in the cobalt ferromagnetic layer, which may influence the Fermi level of FM thin films and then change the magnetic properties.^11^, ^24^, ^25^, ^26^, ^34^, ^35^, ^36^, ^37^ Compared with IL gating method, the optical gating process is a pure physical routine without any possible chemical change and erosive problem.^11^ In addition, this process also applies to flexible electronics/spintronics/photonics for its compatibility with flexible substrates. In device applications, the miniature of the devices is confliction with the dissipation of the current induced thermal heat. By using the photovoltaic control of magnetism, the counterpart control current is not necessary, so that there is no joule heating, and definitely facilitating the miniature of the memory devices.

In this paper, Ta (4 nm)/Co (≈1 nm) was deposited with a magnetron sputtering method onto the Si/SiO_2_ substrate. An organic photoactive semiconductor thin films were spin‐coated onto Co films to form an OME tricoupled bulk heterostructure (BHJ).^38^, ^39^ The optical control of magnetic anisotropy (OCMA) changes was recorded by ESR quantitatively, with 175 and 640.26 Oe ferromagnetic resonance (FMR) field shifting reproducibly and stably under different intensities of illumination, respectively at 50 and 150 mW cm^−2^ in the ambient atmosphere. It should be noticed that there is no photocurrent at the illumination state due to no closed circuit, like a charged capacitor, which is not working to produce current. The first‐principles calculation indicated that the electrostatic doping from the photovoltaic process shifted the Fermi level of Co and reduced the total magnetic strength accordingly. Most importantly, this OME tricoupled work inspires us that two instructive steps could solve the sunlight control of magnetism challenge: finding an effective photoactive semiconductor layer and establishing a charge sensitive magnetic layer. Here, the interfacial photoelectrons overarch the field of solar power and the field of spintronics, creating an interdisciplinary subarea of photovoltaic spintronics.

## 2 Results and Discussion

**Figure 1:**
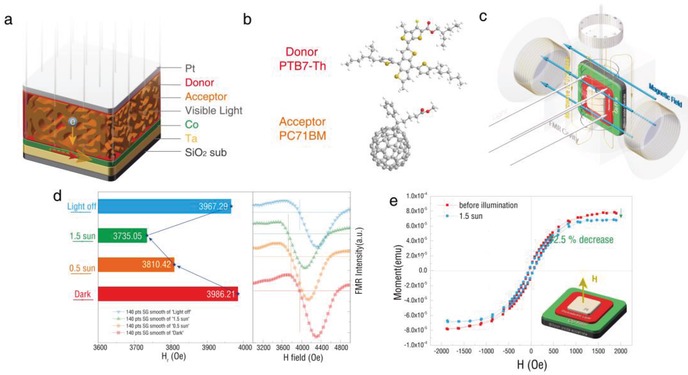
Schematics of visible light photovoltaic gating heterostructure and in situ ESR and VSM measurement of the photovoltaic induces magnetic anisotropy change. a) The Pt/bulk heterostructure/Co/Ta/SiO_2_ photovoltaic gating heterostructure. The visible light (semitransparent white arrow array) excites the organic bulk heterostructure producing electrons. Then the electrons move toward the Co layer, which will induce a magnetic anisotropy change. b) The molecular structure of the donor (PTB7‐Th) and acceptor (PC_71_BM) of the bulk heterostructure. c) The geometry of the in situ ESR measurement, and where the film is parallel to the external magnetic field was defined as 0° as shown in the schematic. d) The photovoltaic‐induced ESR spectrum shift. The red‐square line, orange‐round line, the green‐triangle, and the blue‐invert triangle line stand for the initial state, 0.5 sun gating state, 1.5 sun gating state, and the light‐off state, respectively, which were measured at 90°. e) In situ VSM measurement of the in situ photovoltaic gating.

The schematic of the photovoltaic spintronic heterostructure is shown in **Figure** **1**a. The organic semiconductor layer was spin‐coated on to the Si/SiO_2_/Ta/Co as a light respond layer, where poly[4,8‐bis(5‐(2‐ethylhexyl)thiophen‐2‐yl) benzo [1,2‐b:4,5‐*b*′] dithiophene‐*co*‐3‐fluorothieno [3,4‐*b*] thiophene‐2‐carboxylate] (PTB7‐Th) was used as the donor, and [6,6]‐Phenyl C71 butyric acid methyl ester (PC_71_BM) as the acceptor, on which a 3 nm transparent Pt film was deposited as electrode. Atomic force microscope (AFM) images of the surface about the Si substrate, Co, organic semiconductor layer, and Pt are shown in Figure S1 of the Supporting Information. Figure 1b illustrates the formulas of donor and acceptor molecules. This pair of organic molecules have been proved to be one of the most successful systems of effective excitons separation in organic solar cells (OSCs), and up to 10% power conversion efficiency could be achieved.^40^, ^41^

In situ ESR measurement is utilized to quantify the photo‐induced magnetic anisotropy change of Co. The geometry of in situ photovoltaic spintronic ESR test is demonstrated in Figure 1c, where the angle between the magnetic field and the laminates film was defined as 0° (in‐plane). The magnetic anisotropy analysis method was according to the previous report.^11^ The FMR line illustrated in Figure 1d contain clearly asymmetrical component and possibly multiple FMR peaks, which may be originated from inhomogeneous Co domains in thin film. The standard sunlight intensity is 100 mW cm^−2^ (1 sun). For the Pt/PTB7‐Th:PC_71_BM/Co (0.9 nm) heterojunction, the AM1.5G illumination at 50 mW cm^−2^ induced an −175.79 Oe FMR field (*H*_r_) shift along the out‐of‐plane direction in a reversible manner. During the optical gating process, the thin film of active organic materials absorbed photons to generate excitons, which included a tightly bound electron–hole pair.^29^ Then excitons were separated to form electrons and holes when excitons migrated at the interface of the donor and the acceptor, and the electrons were transferred to the cobalt before being gathered, as illustrated in Figure 1a. Finally, it caused a substantial photon‐induced magnetism modification on Co film through visible light illumination. With a higher illumination power density of 150 mW cm^−2^, the magnetic anisotropy change increased up to −251.16 Oe as shown by the ESR spectrums (Figure 1d), implying that the enhanced separation performance of photoelectrons from vacancies can effectively further weaken the anisotropy of Co layer. Furthermore, vibrating sample magnetometer (VSM) was utilized to investigate the magnetic property change of magnetization (emu cm^−3^) induced by the visible light, as shown in Figure 1e. The samples were tested in the in‐plane direction under 150 mW cm^−2^ illumination. Compared with the initial state, the saturated magnetization decreased by 12.5% under illumination. This is consistent with the ESR measurement as well as the first‐principles calculation, which we will discuss in the later part.

**Figure 2:**
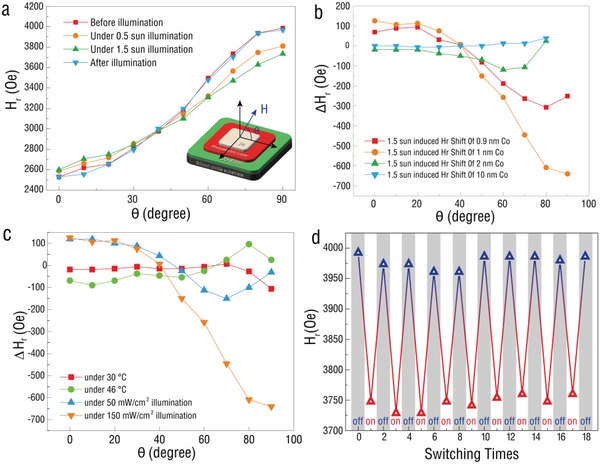
Angular dependence, thickness dependence, and reversibility of the in situ photovoltaic gating ESR measurement. a) Angular dependence of the photovoltaic‐induced ferromagnetic resonance field shift. This shares the same graphic sample with Figure 1d. b) The angular dependence of the FMR field for the 0.9, 1, 2, and 10 nm Co samples, respectively. c) Angular dependence of FMR field variation in situ photovoltaic gating ESR measurement. A comparison among the FMR field for the 1 nm Co sample at RT, 30 and 46 °C, respectively, in darkness and under 0.5/1.5 sun illumination. d) Reversibility test of the in situ photovoltaic gating ESR measurement under multiple fields of magnetism and optic in out‐of‐plane direction under 1.5 sun illumination.

**Figure** **2**a compares the angular dependence of the FMR field shift in the variation of the illumination power density. At the initial state, the in‐plane (0°) was the easy axis due to the smallest *H*_r_ value, and the out‐of‐plane was the hard axis. The increasing light power triggered a weaker magnetic anisotropy of the cobalt layer, evidenced by space magnetic anisotropy information, which was the result of more excitons induced. To exclude the magnetic anisotropy change induced by temperature variation, we conducted a verification experiment. Before the controlled trial, the irradiation‐induced temperature increase was carefully measured. The temperature increased from RT to 30 °C under 50 mW cm^−2^, and to 46 °C under 150 mW cm^−2^, respectively. Correspondingly, the magnetic anisotropy of the same device was in situ tested at RT, 30 and 46 °C in the dark as compared in Figure 2c and Figure S2 (Supporting Information). The FMR fields of the 1 nm Co film at 30 °C (46 °C) both were different from the result of illumination under 0.5 (1.5) sun at different angles, respectively. Compared with the Δ*H*_r_ fields under 0.5 sun intensity of illumination was 119.33 Oe in in‐plane and −30.28 Oe in‐out‐of‐plane, and 125.58 Oe in in‐plane and −640.26 Oe in out‐of‐plane under 1.5 sun intensity of illumination, respectively. The device attained −18.76 Oe in in‐plane and −106.67 Oe in out‐of‐plane at 30 °C, but the *H*_r_ shift can be achieved at −68.97 Oe in in‐plane and +25.18 Oe in out‐of‐plane at 46 °C, respectively. It can be seen that the whole tendency of *H*_r_ changing was different between the heating test and illumination test. This value was not comparable with the −640.26 Oe FMR field shift of Co under 150 mW cm^−2^, which indicated that the light irradiation dominated the magnetic anisotropy manipulation process.

Furthermore, 0.9, 1, 2, and 10 nm of Co layer were used to investigate the thickness‐dependent magnetic anisotropy change under 150 mW cm^−2^ illumination. Δ*H*_r_ is the FMR field shift compared to the “before illumination,” which is expressed as Δ*H*_r_ = *H*_r,illumination_  − *H*_r,dark_. The variation of Δ*H*_r_ and ESR curves with different thickness of Co layer were shown in Figures S3–S10 of the Supporting Information. As shown in Figure 2b, for the 10 nm Co device, FMR fields almost remained constant at different angles. And *H*_r_ shift could be enhanced with the decline of Co thickness around 1 nm due to the weakened shape anisotropy energy, as well as increased charge density for the Co film. Because for a photovoltaic layer, at a settled light intensity, the photogenerated excitations separation rate is constant. The maximal shift of resonance field (−640.26 Oe) was obtained with 1 nm of Co layer. We believed the maximal FMR field shift value did not occur in the 0.9 nm sample because the small variation of the anisotropy field between the in‐plane and out‐of‐plane direction limited the tunable value. Also, Figure 2d demonstrates the reproducible test of the light tuning process with good reversibility in a stable manner. A truly valuable breakthrough to control interfacial magnetism via visible light irradiation is achieved, and it has a potential in new type spintronics based on solar power control.

We believe the FMR field change was achieved via the photovoltaic effect induced electron doping in the cobalt film. To prove this mechanism, first we need to exclude the possible interface coupling effect between the organic semiconductor and the ferromagnetic film as well as the possible magnetic “proximity” effect between the cobalt film and the top Pt capping layer. The coupling between the nonmagnetic layer, such as organic semiconductor, and ferromagnetic layer has been well investigated.^42^, ^43^, ^44^, ^45^ Here, this spin interface effect has been well excluded evidenced by the no difference ESR result between the cobalt film and cobalt film/organic photovoltaic (OPV) layer (Figure S11, Supporting Information). It implies that no magnetic coupling between the PCBM:PTB7‐Th and the cobalt film. From Figure S12a of the Supporting Information, Si/SiO_2_/Co/OPV heterojunction shows the light‐induced FMR field shift phenomena. In addition, the SEM image of heterojunction is shown in Figure S12b of the Supporting Information. The Pt and the photovoltaic layer have a clear boundary. There is no Pt cluster near the Co surface, which demonstrates that there may be no magnetic “proximity” effect between Pt clusters and Co surface. So the magnetic property modulation effect was induced by the photo‐induced electron doping.

**Figure 3:**
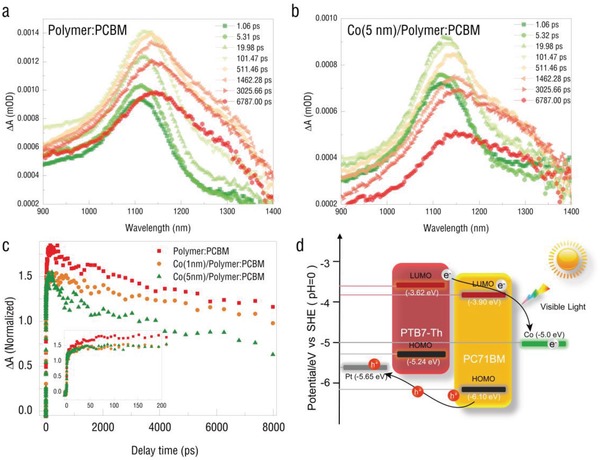
Transient absorption (TA) spectra of PTB7‐Th:PC_71_BM in different media: a) pristine film and b) with Co layer at the indicated delay times. The excitation wavelength is 600 nm with a power of 7 µJ cm^−2^ per pulse. c) The comparison of TA kinetics probed at 1120 nm. d) Schematic diagram of photoinduced electrons transfer route and a simplified energy‐level diagram illustrating the highest occupied molecular orbital (HOMO) and lowest unoccupied molecular orbital (LUMO) energies of donor and acceptor.

To further confirm the charge transfer process from the photovoltaic layer to the magnetic layer, we also carried out the ultrafast transient absorption (TA) measurements on PTB7‐Th:PC_71_BM thin films with and without cobalt layer. The films were excited at 400 nm by a femtosecond pulse laser followed by a probe beam in near infrared radiation (NIR) region from 850 to 1400 nm at various delay time durations. In comparison, TA spectra of PTB7‐Th:PC_71_BM thin films with and without cobalt layer are shown in **Figure** **3**a,b, respectively. Upon excitation, both films exhibited a broad photo‐induced absorption peak centered at ≈1120 nm. This feature is consistent with the results in previous reports and is attributed to the formation of charge‐separated (CS) state in PTB7‐Th:PC_71_BM.^46^, ^47^ For the PTB7‐Th:PC_71_BM/glass sample, the recovery of this signal corresponded to the recombination of the separated electrons (at PC_71_BM) and holes (at PTB7‐Th). However, for PTB7‐Th:PC_71_BM/Co/glass sample, the recovery of this CS state became faster, as illustrated in the comparison of TA kinetics probed at 1120 nm. We attributed the faster recovery kinetics to the transfer of the separated electron from PC_71_BM to the cobalt layer, which is a process dissipating the CS state. More importantly, via the ultrafast TA measurements we ensured that the photovoltaic spintronics response rate was ≈10 ps, as illustrated in Figure 3c. Hence, it shows a new potential in magnetoelectric random access memories and ME sensors.

**Figure 4:**
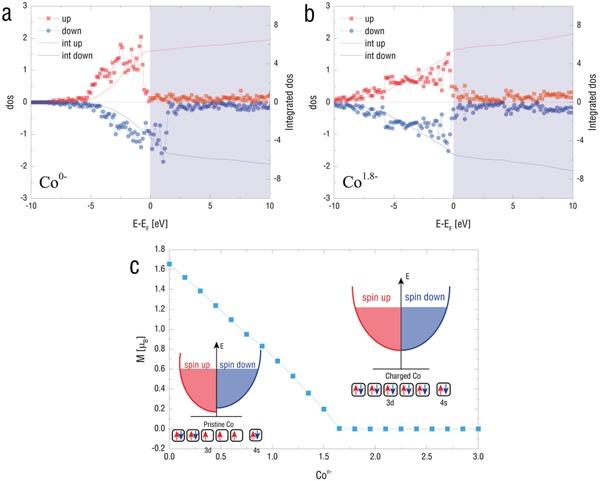
First‐principles calculation of the electrons induced magnetic moment variation. The charge injection‐induced DOS vibration of Co for a) Co^0−^ and b) Co^1.8−^, respectively. c) Moment of the cobalt atom at a function of charge number of the Co^n^. “Int up” and “Int down” mean the integral of the density of the spin‐up state and density of the spin‐down state.

This charge injection process is induced by built‐in electric field, which is caused due to the difference of the work function of the electrodes. During the transferring, photo‐induced electron can be trapped and recombined with hole again. With sunlight, the photoinduced electrons finally accumulate in the magnetic metal electrode (Co) layer. While keeping increasing the electron accumulation at the Co surface, the built‐in electric field becomes weak. Finally, the equilibrium state is obtained where the generation of the electron and the capture of electron is equivalent. Therefore, it will stop or equilibrate when the internal electric field cancels out this difference, resulting from injection charge accumulation. There is no persistent photo‐ induced current because there is no closed loop circuit. The heterostructure at the illumination state is similar to an open‐circuit charged capacitor, not a working battery. The charge doping may cause the magnetic anisotropy change. To reveal the relationship between electron concentration and magnetic moment of Co, we conducted the first‐principles calculation. The charge doping mechanism as illustrated in **Figure** **4**a,b is suppressed by introducing extra electrons via the photovoltaic process, where the original magnetization of cobalt comes from the unpaired electrons inside the 3d band. And in detail, as depicted in Figure 4c, we carried out a continuous electron doping calculation on cobalt, and found that with the increase of doped electrons, the magnetic moment of Co would decrease until it got saturated at Co^1.8−^ state, a state much closer to that of nonmagnetic copper. Considering the experiment process and calculation results, it is evident that the PTB7‐Th:PC_71_BM will change the Co layer under visible light to increase the electron density and to fill the unoccupied orbitals, which end up with an almost equal spin‐up and spin‐down state, and then depress the net magnetization.

Figure 3d demonstrates the mechanism of the optical‐electro‐magnetic coupling effect in the manipulation process of the Pt/PTB7‐Th:PC_71_BM/Co. The simulated sunlight generated excitons at the interface of the PTB7‐Th:PC_71_BM BHJ structure. Based on the different work functions among the Pt, PTB7‐Th, PC_71_BM, and Co, the electrons from the separation of the excitation moved toward and injected into the Co film, and the Fermi Level of the Co was altered with the band filling process. The Co magnetic anisotropy was weakened due to the band filling. The work functions among the Pt, PTB7‐Th, PC_71_BM, and Co were balanced. Meanwhile, the lowered separation rate got to an equilibrium state with the cancellation rate of the electrons and holes. When the light source was switched off, the cancellation rate of the electrons and holes was dominated, resulting in the diminished electron density of the cobalt, and then the magnetic anisotropy change was switched back.

As a consequence, achieving efficient solar control of magnetism is now divided into two major missions: first, finding powerful photoelectric media that can provide a large number of photoelectrons toward photoelectric/magnetic interface, and second, establishing ferromagnetic thin films and heterojunctions that are sensitive to photoelectric doping electrons. Splitting one complex challenge into two simpler ones accelerates the solution of the problem. Compared with traditional direct photon‐spin coupling approaches, the OME heterojunction strode the complex physics mechanism and simplified the task into two engineering questions.^21^ Thus, the booming of photovoltaic spintronics could be highly expected in many other spintronic systems, for instance, visible light controllable magnetic heterojunctions, topological spintronics, and flexible spintronics.

## 3 Conclusion

In summary, the photovoltaic spintronics device was fabricated successfully with the structure of Co/organic active layer/Pt. Because the excitons were generated in the organic semiconductor BHJ structure (PTB7‐Th:PC_71_BM) and then photoelectrons were transferred to Co, the diverse ferromagnetic resonance field variation on the Co layer under different intensities of visible light was obtained. About 640.26 Oe of maximal FMR shift was also achieved. On the other hand, the saturated magnetization was decreased by 12.5% under illumination by in situ VSM measurement. The first‐principles calculation was also conducted to reveal the relationship between electron concentration and magnetic moment of Co. The device demo testing showed the good reversibility of magnetism under the introduced light field. Hence, the booming of photovoltaic spintronics may be highly expected in various spintronic systems in the future.

## 4 Experimental Section

*Fabrication and Characterization of the Photovoltaic Spintronic Device*: The device diagram of photovoltaic spintronics was SiO_2_/Si/Ta (4 nm)/Co (*x* nm)/PTB7‐Th:PC_71_BM (54 nm)/Pt (3 nm). The Co (*x* = 0.9, 1, 2, and 10 nm) films were deposited onto the substrates with a DC magnetron sputtering at room temperature as bottom electrodes. In the coating process, the film thickness was controlled with quartz crystal microbalance integrated into the magnetron sputtering system. No further in situ annealing was carried out. PTB7‐Th (D) and PC_71_BM (A) were purchased from 1‐Material Chemscitech Inc. (Canada) and used as received. Active layer solutions (D/A ratio 1:1.5) with polymer concentrations of 8 mg mL^−1^ were prepared in halogen free solvent (*o*‐xylene) with 3% (volume fraction) of 1,8‐diiodooctane (DIO). The solution was stirred overnight at 80 °C before fabrication of the active organic layer. Active layers were spin‐coated by the polymer solution on the substrate in an ambient atmosphere at 1500 rpm to obtain the thicknesses of ≈70 nm. The samples coated with 3 nm Pt were deposited as the top electrode.

In situ magnetic anisotropy modification was carried out in the ESR spectra (JES‐FA200, JEOL RESONANCE Inc.), the rotator of which can show the angle between the film plane and the applied magnetic field. The TE 011 mode microwave power by the microwave unit was 9200 MHz. Devices were illuminated under AM1.5G (100 mW cm^−2^) using a PL‐XQ500W Xenon lamp solar simulator.

*Ultrafast Transient Absorption Spectroscopy Measurement*: The femtosecond transient absorption setup of this study was based on a regenerative amplified Ti:sapphire laser system from Coherent (800 nm, 35 fs, 6 mJ per pulse, and 1 kHz repetition rate), nonlinear frequency mixing techniques, and the Helios ultrafast transient absorption spectrometer (Time‐Tech Spectra, femtoTA100Ultrafast Systems LLC). Briefly, the 800 nm output pulse from the regenerative amplifier was split into two parts with a 50% beam splitter. The transmitted part was used to pump a TOPAS optical parametric amplifier (OPA), which generated a wavelength‐tunable laser pulse from 250 nm to 2.5 m. Here a 400 nm laser was used as a pump beam. The reflected 800 nm beam was split again into two parts. One part with less than 10% was attenuated with a neutral density filter and focused into a YAG window to generate an NIR light from 850 to 1500 nm used as probe beam. The probe beam was focused with an Al parabolic reflector onto the sample. After the sample was settled, the probe beam was collimated and then focused into a fiber‐coupled spectrometer with CMOS sensors and detected at a frequency of 1 KHz. The delay between the pump and probe pulses was controlled by a motorized delay stage. The pump pulses were chopped by a synchronized chopper at 500 Hz, and the absorbance change was calculated with two adjacent probe pulses (pump‐blocked and pump‐unblocked).

## Conflict of Interest

The authors declare no conflict of interest.

## Supporting information

Supplementary
