# Supplementary material for: Sunlight Control of Interfacial Magnetism for Solar Driven Spintronic Applications
Source: Adv Sci (Weinh). 2019 Oct 26;6(24):1901994. doi: 10.1002/advs.201901994 (PMC6918118; doi:10.1002/advs.201901994)
Supplement: Supplementary file 1 — Supplementary [file ADVS-6-1901994-s001.pdf]

Copyright WILEY-VCH Verlag GmbH & Co. KGaA, 69469 Weinheim, Germany, 2019.

Supporting Information

for *Adv. Sci.,* DOI: 10.1002/advs.201901994

Sunlight Control of Interfacial Magnetism for Solar Driven Spintronic Applications

Yifan Zhao, Shishun Zhao, Lei Wang, Ziyao Zhou, Junxue Liu, Tai Min, Bin Peng, Zhongqiang Hu, Shengye Jin, and Ming Liu**

((Supporting Information can be included here using this template))

Copyright WILEY-VCH Verlag GmbH & Co. KGaA, 69469 Weinheim, Germany, 2019.

Supporting Information

Sunlight Control of Interfacial Magnetism for Solar Driven Spintronic Applications

Yifan Zhao,[1,2,#] Shishun Zhao,[1,#] Lei Wang,[3,#] Ziyao Zhou,[*] Junxue Liu,[4] Tai Min, [3] Bin Peng, [1] Zhongqiang Hu, [1] Shengye Jin,[4] Ming Liu[1,2,]*

1. Electronic Materials Research Laboratory, Key Laboratory of the Ministry of Education & International Center for Dielectric Research, School of Electronic and Information Engineering, and State Key Laboratory for Mechanical Behavior of Materials, Xi'an Jiaotong University, Xi'an 710049, China

2. International Joint Laboratory for Micro/Nano Manufacture and Measurement Technology, Xi'an Jiaotong University, Xi'an 710049, China

3. Center for Spintronics and Quantum System, State Key Laboratory for Mechanical Behavior of Materials, School of Materials Science and Engineering, Xi'an Jiaotong University, Xi'an, Shaanxi, 710049, China

4. State Key Laboratory of Molecular Reaction Dynamics and Collaborative Innovation Center of Chemistry for Energy Materials (iChEM), Dalian Institute of Chemical Physics, Chinese Academy of Sciences, 457 Zhongshan Rd., Dalian, 116023, China.

# These authors contributed equally to this work.
*E-mail: mingliu@xjtu.edu.cn

Supplementary Experiment

1.AFM Analysis for the Suface of Each Layer of Photovoltaic Spintronic Device

The Si substrate, Co (0.9 nm) layer and organic photovoltaic (OPV) active layer with/without top electrode of Pt (3 nm) is observed via AFM as illustrated in Figure S1(a)~(d), respectively. The height images of Si substate was shown in Figure S1 (a), and it exhibited a very flat surface of Si. The room means square (RMS) roughness of the Si substrate is 0.34 nm. The same consequence was got after Co layer was deposited on to the substrate as shown in Figure S1 (b), and RMS roughness was declined to 0.25 nm after Co was fabricated on the Si substrate. The

surface morphology was rough after the OPV active layer was spin-coated and the RMS roughness was achieved to 1.29 nm. It remained to decline to 1.28 nm after Pt was deposited. It is suggested that 3 nm Pt layer didn't increase the surface roughness, and it forms the continuous thin film as the top electrode.

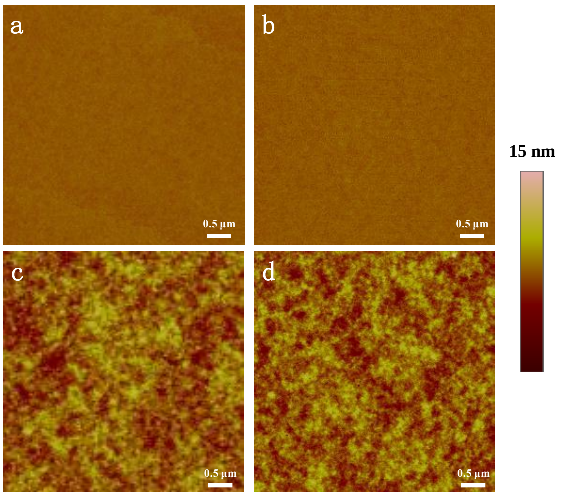

Figure S1. AFM height images of the top surface of sample (a) Si/SiO_2 substrate, (b) Co layer on Si/SiO_2 substrate, (c) OPV / Co / Si/SiO_2 and (d) Pt/ OPV / Co / Si/SiO_2layer. All the images' scale is 5 µm × 5 µm

2. ESR test of Magnetic Anisotropy Change under Temperature Effect

Temperature variation can be induced by illumination of visible light. Therefore, the verification test was also performed. To quantitatively determine the change of the temperature under sunlight, a thermocouple was supposed to get contacted with the sample in ESR cavity during the measurement. However, loading a thermocouple into the cavity enables a huge microwave loss so that the magnetic signal is too weak to be detected. Therefore, we carried out a control experiment to evaluate the temperature effect.

In the beginning, a thermometer was used to precisely determine the maximum temperature change under a certain illumination intensity. To do so, the bulb of the thermometer was blackened prior to the test. During the evaluation, the irradiation time was long enough to ensure the temperature was stable. On the other hand, the light spot focused on the sample

surface is large enough to cover the sample device so that the sample temperature can be identified as the surrounding temperature. The temperature vibration test was in situ conducted in the ESR cavity with air flow heating. By comparing the angular dependence FMR field results of the samples from the light illumination test and the heating test, the heating effects can be excluded. Finally, we found the temperature was changed from room temperature to 30 ^{o}C with illumination of 50 mW cm^{-2} and 46 ^{o}C with illumination of 150 mW cm^{-2}, respectively. Similarly, the temperature was set as 30 ℃ and 46 ℃ in in-situ ESR measurement, respectively.

We assume that the sample also undergoes such thermal influence with sunlight illumination. The temperature change may be equal or less than the control experiment as described above, which is determined to be 30 ^{o}C (50 mW cm^{-2}) and 46 ^{o}C (150 mW cm^{-2}). In order to investigate the pure thermal effect on the magnetic properties of the sample, we adjusted the cavity and sample temperature to 30 ^{o}C and 46 ^{o}C carried out ESR test without sunlight illumination.

An apparent opposite trend is observed as illustrated in **Figure S2**. When the external magnetic field is along the in-plane direction, the resonance field keeps increasing under various sunlight intensity at room temperature. However, without illumination, the resonance field decreases as increasing the sample temperature. These results indicate the pure heating effect and photovoltaic effect shift H_r oppositely. The same result is obtained when the external magnetic field is along the out-of-plane direction. In our case, the photovoltaic effect is much more dominated which overcomes the thermal effect and shifts H_r upwards. Therefore, the heating effect can be excluded and the total Hr shift is attributed to the photovoltaic effect.

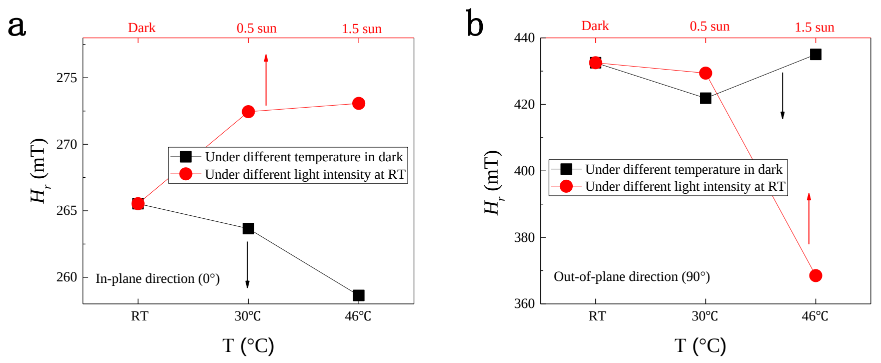

Figure S2. FMR field variation at a certain angle under photovoltaic effect and temperature effect via ESR measurement, respectively. (a) in-plane direction (at 0°), (b) out-of-plane direction (at 90°).

3. Variation of ΔH_r under Different Intensities of Illumination with 1, 2, 10 nm Co layer.

The devices with different thickness of the Co layer were also fabricated and tested. The results of the angular dependence of the photovoltaic induced FMR field shift were shown in Figure S3, S4, and S5, respectively. From Figure S3, there is a distinct variation of FMR field under the different illumination of visible light. However, the FMR field shift is hardly observed with the thickness of Co increasing. As shown in Figure S4 and S5, the field shift of FMR cannot be obtained with 2 nm and 10 nm Co layer.

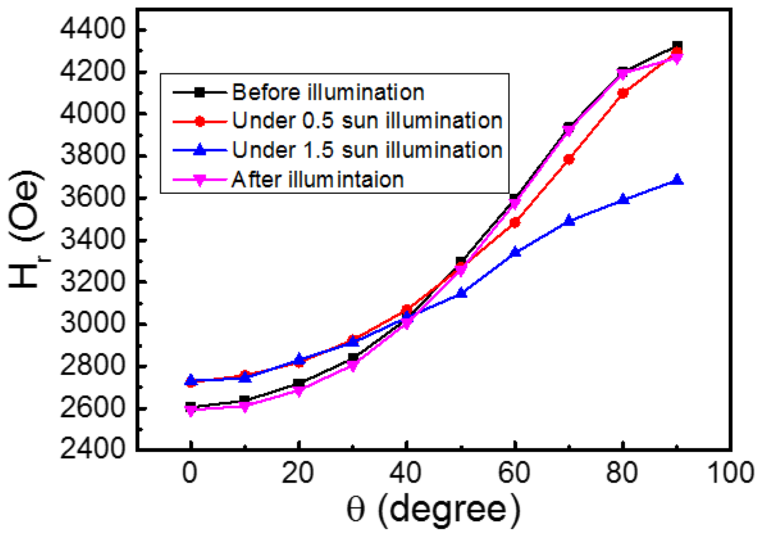

Figure S3. Angular dependence of the photovoltaic induced ferromagnetic resonance field shift with 1 nm Co layer.

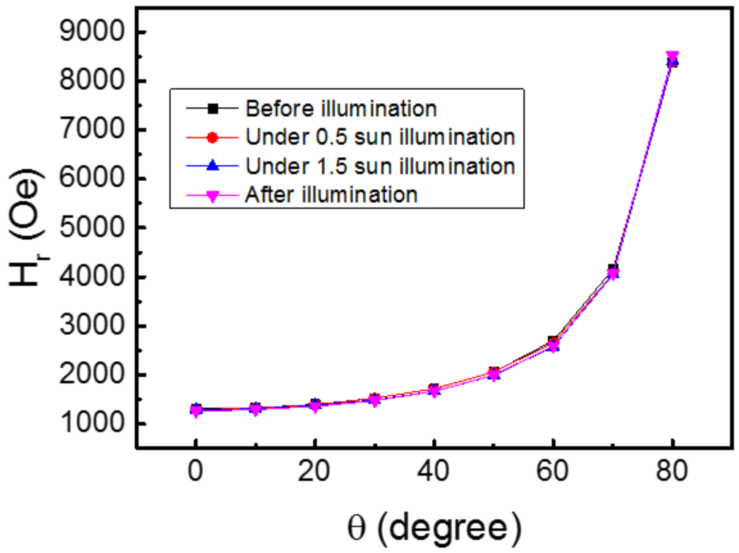

Figure S4. Angular dependence of the photovoltaic induced ferromagnetic resonance field shift with 2 nm Co layer.

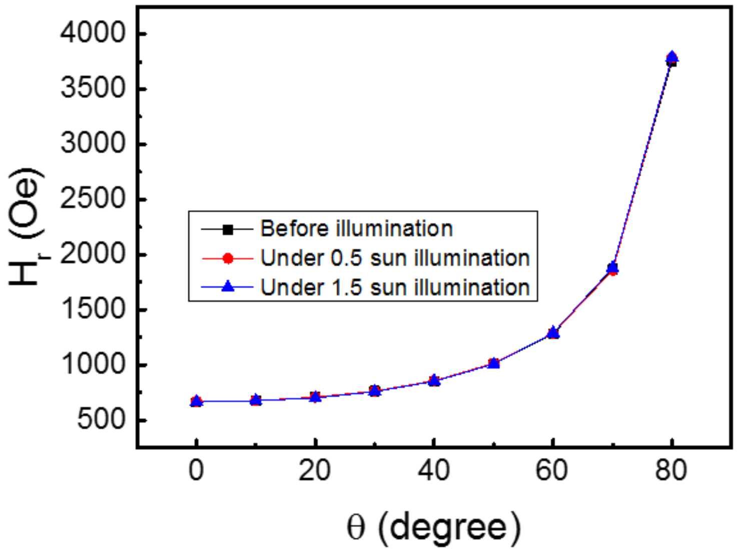

Figure S5. Angular dependence of the photovoltaic induced ferromagnetic resonance field shift with 10 nm Co layer.

4. ESR test of Magnetic Anisotropy Change under Different Intensities of Illumination with 0.9, 1, 2, 10 nm of Co Layer.

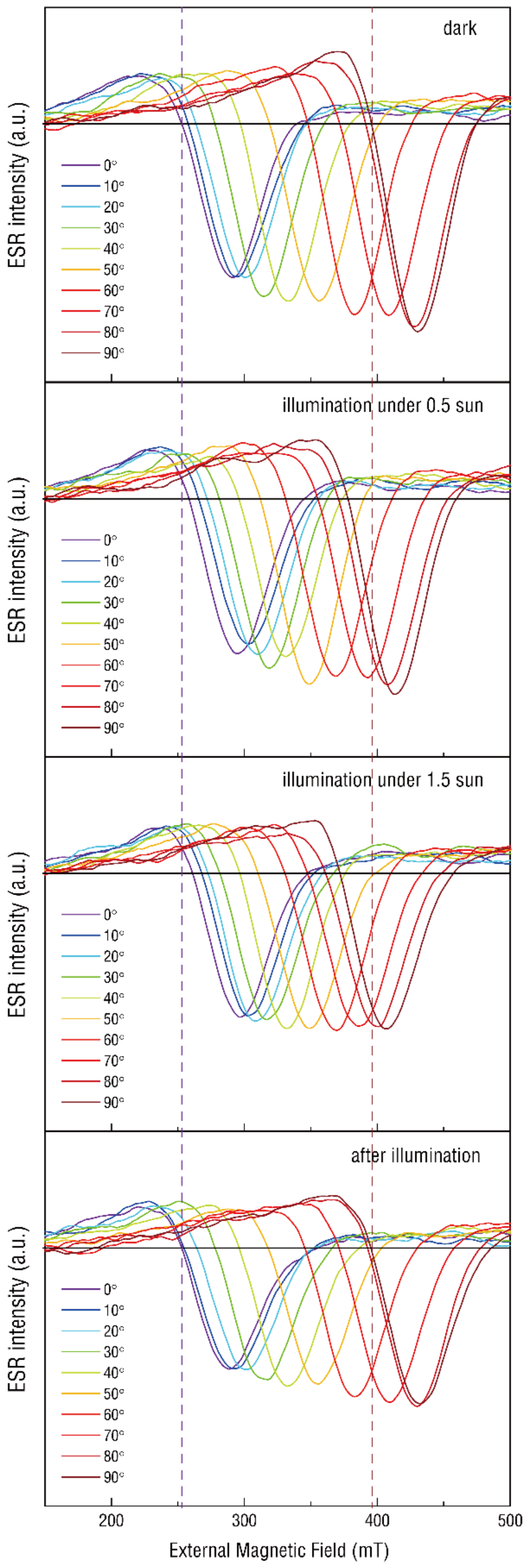

Figure S6 ESR spectrums with in situ photovoltaic gating with 0.9 nm Co; the spectrums were plotted after 140 pts smooth.

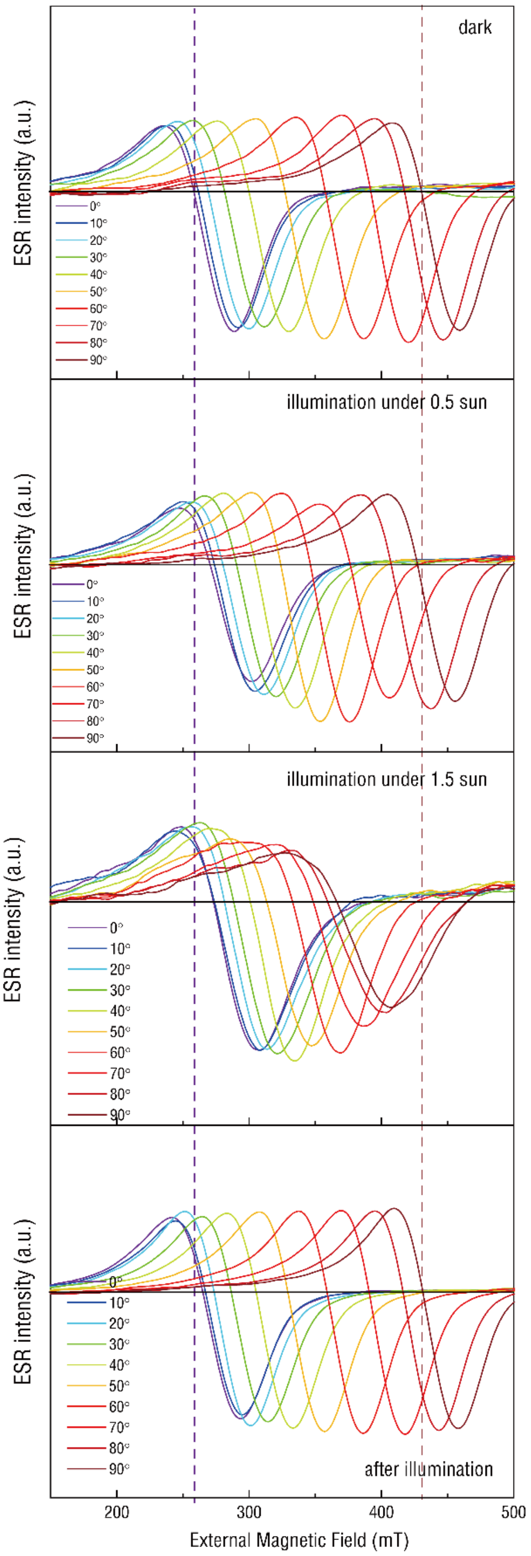

Figure S7 ESR spectrums with in situ photovoltaic gating with 1 nm Co, and the spectrums were plotted after 140 pts smooth.

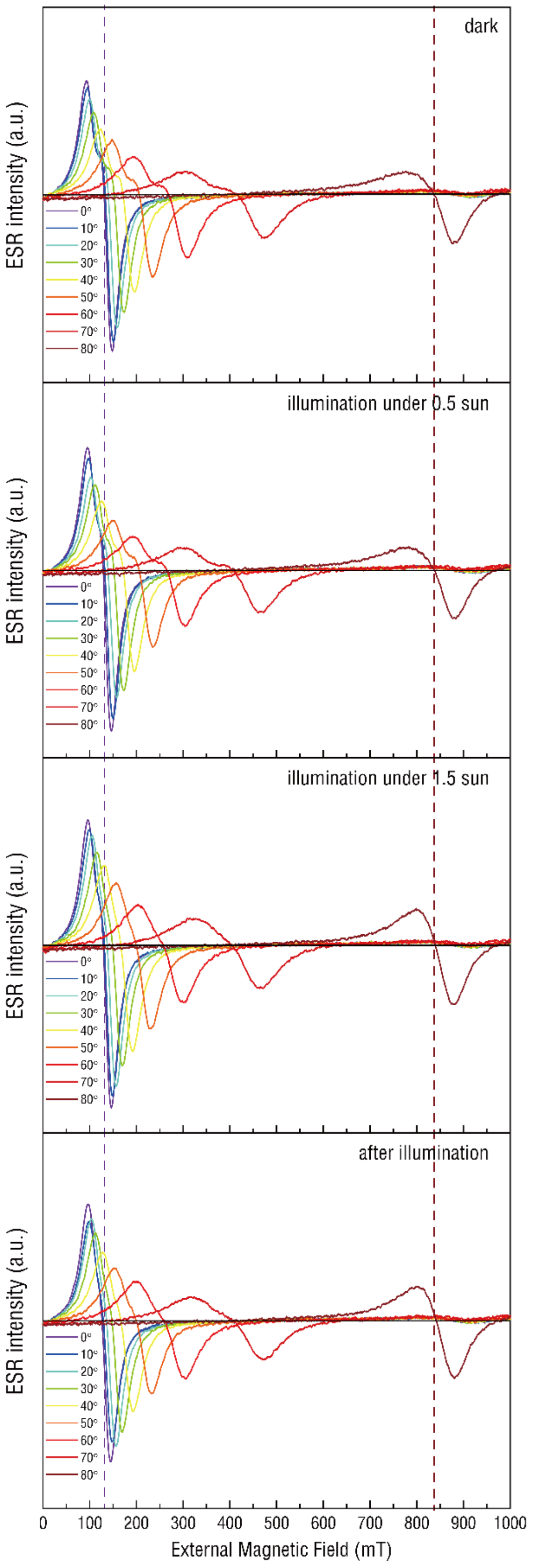

Figure S8 ESR spectrums with in situ photovoltaic gating with 2 nm Co; the spectrums were plotted after 140 pts smooth.

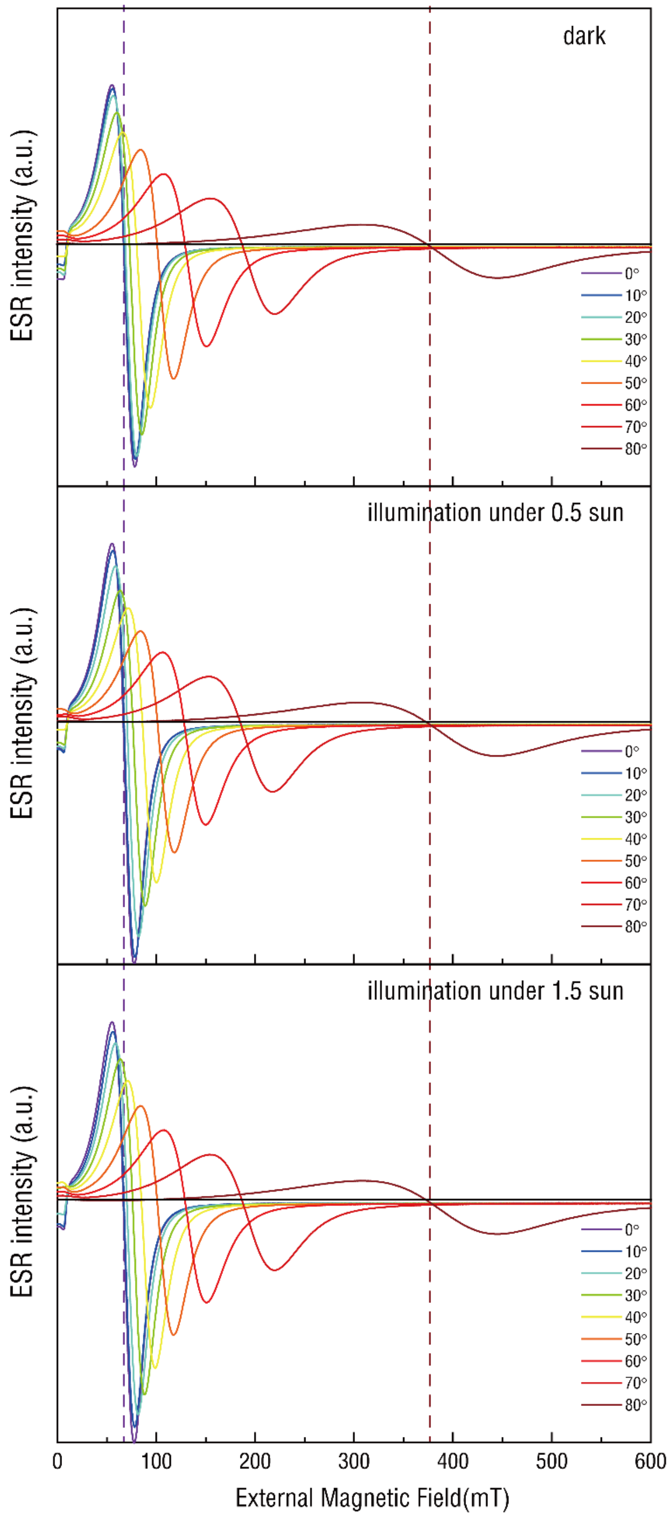

Figure S9 Angular dependence of in situ of the ESR with photovoltaic gating with 10 nm Co, and the spectrums were plotted after 140 pts smooth.

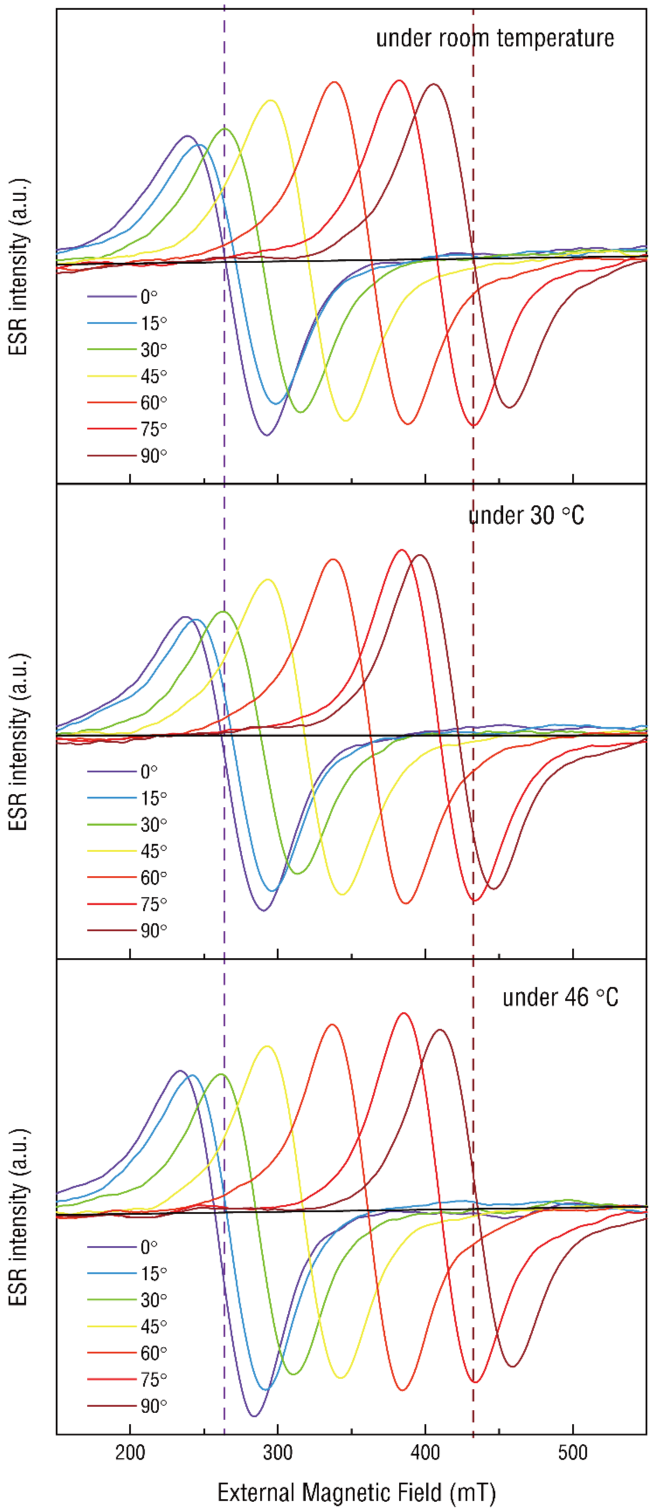

Figure S10 ESR spectrums with in situ photovoltaic gating with 0.9 nm Co; the spectrums were plotted after 140 pts smooth.

5. ESR test of the coupling between the nomagnetic layer and the cobalt film.

To distinguish our work from the coupling between the photovoltaic layer and Co layer, we conducted an additional experiment to see the magnetic property difference between the as grown Co film, and the Co film sample coated photovoltaic layer (PCBM: PTB7-Th) as illustrated in Figure S11.

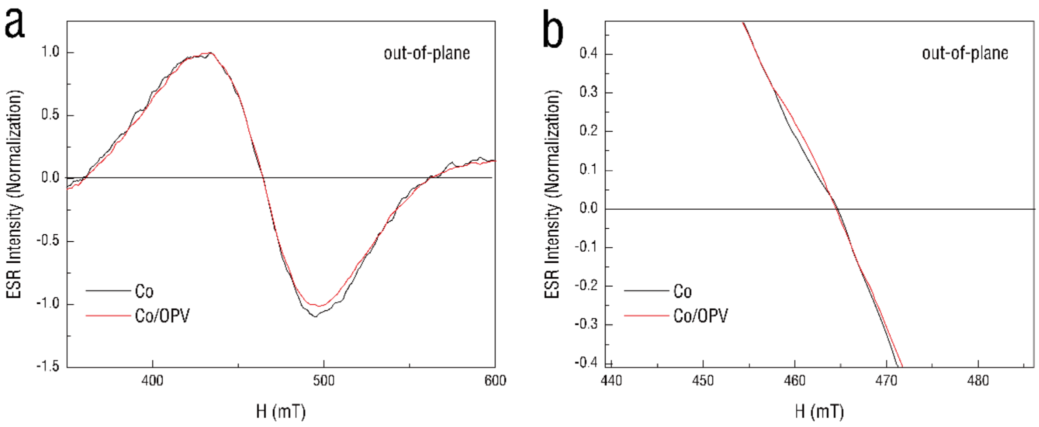

Figure S11 (a) ESR spectrums of the as grown Co film and the Co film sample coated photovoltaic layer (PCBM: PTB7-Th), respectively. (b) gives out the zoom in view of (a).

6. ESR test of the Si/SiO2/Co/OPV under illumination and SEM image of cross section view of Si/SiO2/Co/OPV/Pt structure.

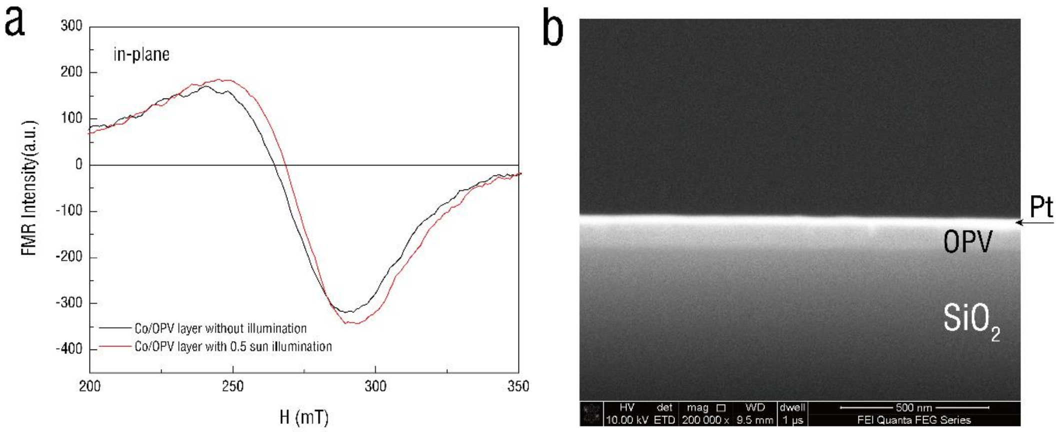

Figure S12 (a) Si/SiO2/Co/OPV also shows the light induced FMR field shift phenomena; (b) Cross section view of the Si/SiO2/Co/OPV/Pt heterostructure from SEM.

7. Confirmatory experiments on the direction of electron migration

The following experiment has proved the direction of photo-induced electrons migration. The manufacturing process is illustrated in Figure S13(a)~(d) and the schematic diagram of test heterostructure is shown in Figure S13(e). The active area of the device is 5 mm × 5 mm. The 2182A NanoVoltmeter was used to detect the variation of voltage difference caused by visible light illumination. The results of the voltage were shown in Figure S14. The Co and Pt layer were connected with the positive and negative probe of NanoVoltmeter, respectively. Generally, complete darkness condition cannot be achieved in an environmental atmosphere so that the NanoVoltmeter can collect data of voltage because of ambient light illumination. Therefore, -0.0184 mV and 0.0169 mV were recorded without visible light illumination as illustrated in Figure S13(b) and (e), respectively. At first, as shown in Figure S13(a), the Co was connected with the positive probe, and Pt was connected with the negative probe of NanoVoltmeter. The voltage detection data was shown with a minus sign in Figure S13(b) and (c). The voltage was enhanced from -0.0184 mV to -2.216 mV under the 1.5 sun illumination. It is demonstrated that the photo-induced electrons can be diffused indeed to the Co layer under the visible light illumination. Then, the minus sign in voltage detection data was removed after the positive and negative probe were exchanged to Pt and Co showed in

Figure S13 (d)~(e), respectively. Also, the voltage variation under 1 sun illumination was consistent with the consequence under 1.5 sun illumination. A video is available as supplementary materials.

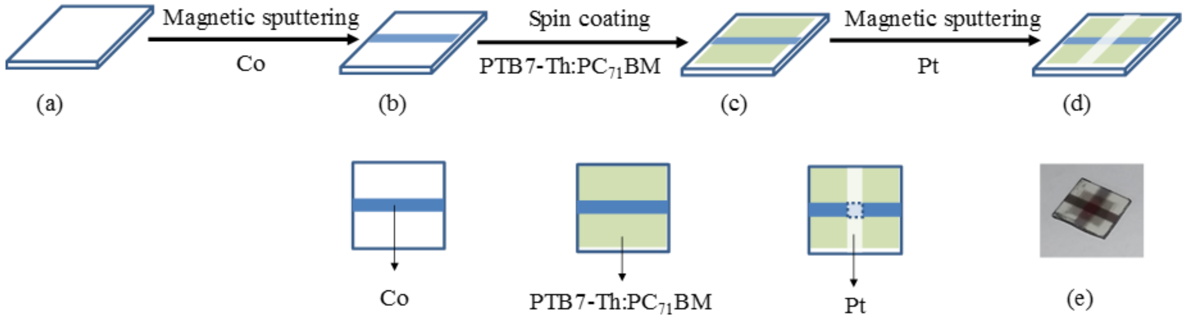

Figure S13. The manufacturing and preparation process of the device for verification. (a) Glass substrate. (b) Co was deposited by a magnetic sputtering process. (c) The active layer was deposited by a spin coating process. (d) A magnetic sputtering process deposited Pt layer. (e) The picture of photovoltaic spintronics device.

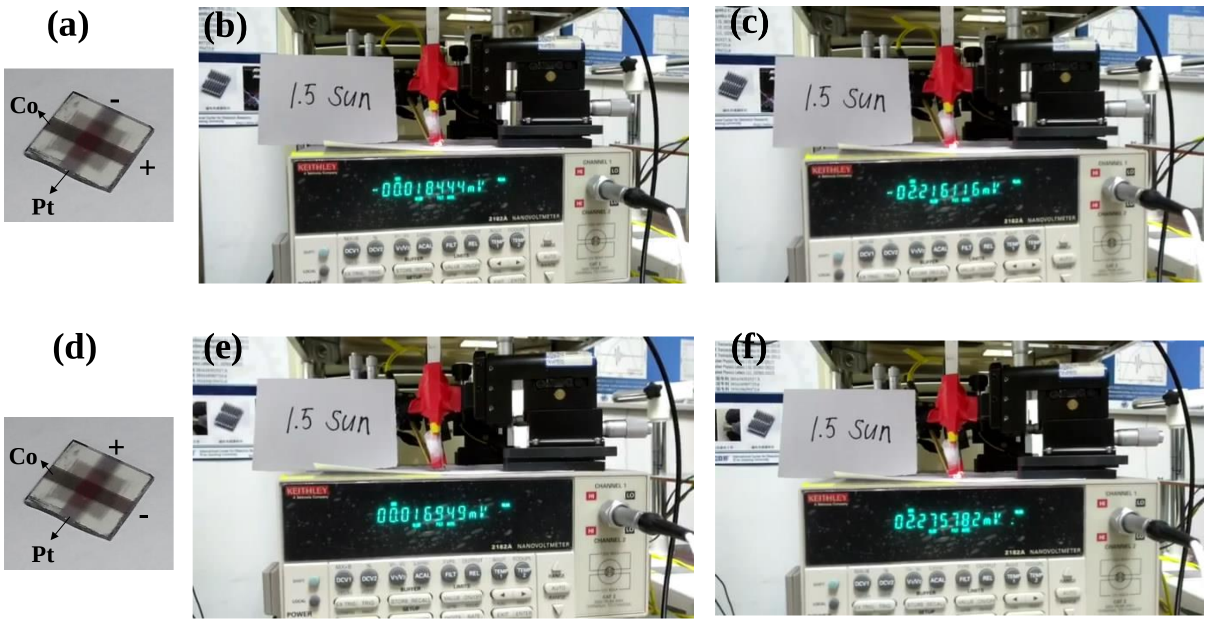

Figure S14. (a) and (d)Positive and negative probe connection with Co and Pt, respectively. (b) and (e) The voltage data with/without minus sign without light illumination. (c) and (f) The voltage data with/without minus sign under light illumination.

The work function of Co and Pt is 5 eV and 5.65 eV, respectively. The device structure of this work is Co/photovoltaic active layer/Pt, which is a classic concept metal-insulator-metal (MIM). And it is also a typical constructer of organic solar cells (OSCs), which is a well-known structure in solar cell community. The metal (Co) was chosen as the electrode as well as the magnetic layer. When the device is in the light soaking, the holes and electrons move to the anode and cathode with driving forces, respectively, rather than the random hopping of the excitons. In the beginning, a gradient in the potentials of electrons and holes exists in a donor-acceptor junction, which is determined by the offset of HOMO of the donor and the LUMO of the acceptor. This internal electrical field determines the maximum open circuit voltage and drives the movement of charge carriers. Then, an external electric field can be formed by using asymmetrical contacts where one low work-function (Co 5 eV) metal is for collecting electrons and one high work-function metal (Pt 5.65 eV) for collecting holes.[1, 2] Finally, the concentration gradients of the positive and negative charges can be formed, respectively. After the charge carriers transport to the interface of the organic materials and electrodes, they are extracted to the electrodes. An ohmic contact between organic materials and electrodes is important to efficiently collect the electrons in cathode and holes in the anode. In our work, in PTB7-Th: $PC_{71}BM$ system Pt with a work function of 5.65 eV was used to match the HOMO of PTB7-Th while low work function metals such as Co (5 eV) was used to match the LUMO of $PC_{71}BM$.

8.Detail of the first principle calculations

All the calculations are performed by the first-principles calculation method based on the density functional theory (DFT) as implemented in the Vienna ab initio simulation package (VASP) [3, 4]. The generalized-gradient-approximation (GGA) with an interpolation formula according to Vosko, Wilk and Nusair [5] and a plane-wave basis set within the framework of the projector augmented wave (PAW) method [6,7] were employed. The cutoff energy for the

basis was 500 eV, and the convergence criterion for the electron density self-consistency cycles was 10−6 eV per atom. In the Brillouin zone, we sampled (15 × 15 × 15) k-point grids using the Monkhorst−Pack scheme [8] to make sure the results converged.

References

[1] I. D. Parker, *Journal of Applied Physics* **1994**, 75, 1656.
[2] V. Coropceanu, J. Cornil, D. A. da Silva Filho, Y. Olivier, R. Silbey, J.-L. Bredas, *Chemical Reviews* **2007**, 107, 2165.
[3] G. Kresse and J. Hafner, *Phys. Rev. B* **1993**, 47, 558.
[4] G. Kresse and J. Furthmüller, *Phys. Rev. B* **1996**, 54, 11169.
[5] S. H. Vosko, L. Wilk and M. Nusair, Can. *J. Phys.* **1980**, 58, 1200.
[6] Blöchl, P. E. Phys. Rev. B: Condens. *Matter Mater. Phys*. **1994**, 50, 17953−17979.
[7] Kresse, G.; Joubert, D. *Phys. Rev. B*: Condens. Matter Mater. Phys. **1999**, 59, 1758−1775.
[8] Monkhorst, H. J.; Pack, J. D. *Phys. Rev. B* **1976**, 13, 5188−5192.
